# A Systematic Exploration of B–F Bond Dissociation Enthalpies of Fluoroborane-Type Molecules at the CCSD(T)/CBS Level

**DOI:** 10.3390/molecules28155707

**Published:** 2023-07-28

**Authors:** Robert J. O’Reilly, Amir Karton

**Affiliations:** School of Science and Technology, University of New England, Armidale, NSW 2351, Australia

**Keywords:** CCSD(T), W1 theory, density functional theory, bond dissociation enthalpies, fluoroborane, boron fluorides

## Abstract

Fluoroborane-type molecules (R^1^R^2^B–F) are of interest in synthetic chemistry, but to date, apart from a handful of small species (such as H_2_BF, HBF_2_, and BF_3_), little is known concerning the effect of substituents in governing the strength of the B–F bonds of such species toward homolytic dissociation in the gas phase. In this study, we have calculated the bond dissociation enthalpies (BDEs) of thirty unique B–F bonds at the CCSD(T)/CBS level using the high-level W1w thermochemical protocol. The B–F bonds in all species considered are very strong, ranging from 545.9 kJ mol^−1^ in (H_2_B)_2_B–F to 729.2 kJ mol^−1^ HBF_2_. Nevertheless, these BDEs still vary over a wide range of 183.3 kJ mol^−1^. The structural properties that affect the BDEs are examined in detail, and the homolytic BDEs are rationalized based on molecule stabilization enthalpies and radical stabilization enthalpies. Since polar B–F bonds may represent a challenging test case for density functional theory (DFT) methods, we proceed to examine the performance of a wide range of DFT methods across the rungs of Jacob′s Ladder for their ability to compute B–F BDEs. We find that only a handful of DFT methods can reproduce the CCSD(T)/CBS BDEs with mean absolute deviations (MADs) below the threshold of chemical accuracy (i.e., with average deviations below 4.2 kJ mol^−1^). The only functionals capable of achieving this feat were (MADs given in parentheses): ωB97M-V (4.0), BMK (3.5), DSD-BLYP (3.8), and DSD-PBEB95 (1.8 kJ mol^−1^).

## 1. Introduction

The chemistry of the boron trifluoride (BF_3_) and the so-called fluoroborane (R^1^R^2^BF) and difluoroborane (RBF_2_) derivatives are of relevance to both industrial and synthetic organic chemistry [1]. Beginning with BF_3_, which is perhaps the most widely studied of these compounds, a diverse range of reactions involving this molecule have been reported. From the perspective of its reaction in the gas phase, representative examples of such studies include its reaction with (i) catecholate and related anions [2], (ii) the gas phase ion chemistry of BF_3_/CH_4_ mixtures [3], and (iii) the reaction of ammonia and methylamine [4,5]. From the perspective of the reactions of BF_3_ in solution (where it is often used in the form of its etherate adduct), it has been shown to play a role in facilitating various polymerization reactions [6,7,8], dehydration reactions of alcohols [9,10], esterification reactions [11,12,13], alkylation reactions [14,15], as well as the synthesis of *syn*-fluorohydrins from epoxides [16]. It has also been reported that triplet diphenylcarbene could be inserted into the B–F bond of BF_3_ [17].

Turning our attention now to monofluoroboranes (i.e., of the type R^1^R^2^BF), we note that several such species have been synthesized previously. The prototypical fluoroborane, BH_2_F, has been detected using microwave spectroscopy [18], and using the experimental ground-state rotational constants and ab initio vibration-rotation coupling constants, an accurate geometry of H_2_BF has been determined [19]. In addition, by way of microwave spectroscopy, the adduct formed between the interaction of H_2_BF and trimethylamine has also been investigated spectroscopically [20]. A particularly well-studied monofluoroborane, which is commercially available, is dimesitylboron fluoride (Mes_2_BF), for which a crystal structure has been obtained [21]. This reagent has been used in, for example, the synthesis of *o*-carborane-substituted triarylboranes [22] and also the synthesis of a series of two-coordinate and quasi-two-coordinate transition metal complexes [23]. Another reagent that has broader potential use in synthetic chemistry is pinacolatoboron fluoride (pinBF), which was shown to be an efficient fluoride transfer agent for the diastereoselective synthesis of benzylic fluorides [24]. In addition, fluorodihydroxy borane, BF(OH)_2_, has also been synthesized in the gas phase [25]. We also note that numerous difluoroborane species (i.e., RBF_2_) have also been produced previously. The prototypical molecule HBF_2_ has been synthesized [26], while several alkyl-substituted difluoroboranes [27], vinyl [28], and aryl-substituted [29,30] species have also been prepared. In addition to carbon-based substituents, we also note that the synthesis of aminodifluoroborane (H_2_NBF_2_) has also been reported, and its IR [31] and microwave spectra [32] have been obtained and analyzed.

To better understand the thermodynamic stability of fluoroborane-type species, it would be insightful to have a greater understanding of the effect that substituents play in governing the strength of the B–F bonds toward homolytic cleavage (i.e., the energies associated with Equation (1)).

R^1^R^2^B–F → R^1^R^2^B^•^ + F^•^ (1)

To date, the only reliable data that have been reported concerning the gas-phase homolytic B–F BDEs of such species are limited to the BH_2_F, BHF_2_, and BF_3_. The first study reporting the BDEs of these species, which was reported by Rablen and Hartwig, used the more approximate G2 and CBS-4 protocols [33]. A second and more recent study, which utilized a more robust level of theory, in particular, a layered extrapolation to the all-electron relativistic CCSD(T)/CBS level of theory (coupled-cluster with single, double, and perturbative triple excitations at the complete basis set limit), was reported by Grant and Dixon [34]. The results of this investigation provided B–F BDEs (at 0 K) for these three molecules of 703.7 (BH_2_F), 721.7 (HBF_2_), and 712.5 (BF_3_) kJ mol^−1^. By any measure, the fact that only three homolytic gas-phase B–F BDEs of fluoroborane-type molecules have been obtained using reliable thermochemical methods clearly constitutes a void regarding our knowledge of the broader extent to which substituents affect the strength of B–F bonds toward homolytic dissociation.

To address the lack of a broad survey of the effect of substituents in governing the strength of B–F bonds toward homolytic dissociation, the present study reports a set of thirty gas-phase B–F BDEs obtained using the benchmark-quality W1w thermochemical protocol [35], which constitutes a layered extrapolation to the all-electron relativistic CCSD(T)/CBS energy [36]. Furthermore, using the thirty B–F BDEs obtained using the W1w thermochemical protocol as reference values, we have also assessed the performance of a plethora of DFT methods (in conjunction with the A′VQZ basis set) for their ability to compute accurate B–F BDEs. 

## 2. Results and Discussion

### 2.1. Insights Concerning the Performance of B3LYP/A′VTZ for Obtaining the Geometries of Fluoroborane-Type Molecules

We begin by making some comparisons between various geometric parameters for several fluoroborane-type molecules and fluoroboryl radicals, optimized at the B3LYP/A′VTZ level of theory, versus the corresponding values obtained previously using the more rigorous the CCSD(T)/aug-cc-pVTZ level of theory (abbreviated as CCSD(T)/aVTZ) [34]. The geometric parameters for these species obtained at these two levels of theory are provided in Table 1.

For this set of molecules, we note that there is generally good agreement between the values obtained at the B3LYP/A′VTZ level of theory and those obtained by way of higher-level CCSD(T)/aug-cc-pVTZ calculations. This finding is consistent with a recent benchmark study, in which it was noted that the B3LYP functional was found to perform well for the computation of equilibrium bond lengths when assessed against a dataset containing a diverse array of 246 different bond types (including a number of B–H, B–F, and B–C bonds), attaining a root-mean-square deviation (RMSD) of 0.0059 Å [37]. In the context of the present study, and beginning with H_2_BF, we note that both CCSD(T) and B3LYP afford the same value for the H–B–F angle (117.8°), while there is also good agreement between the two levels of theory concerning the B–H and B–F bond lengths, which differ by just 0.002 Å and 0.003 Å, respectively. In addition, for H_2_BF, it is possible to make a comparison between the theoretically determined values and those obtained via experiment [19]. In this regard, we note that the theoretically determined bond lengths are in good agreement with the experimentally reported *r_e_* values of 1.1891(3) and 1.3155(2) Å for the B–H and B–F bonds, respectively. Turning our attention now to the structure of difluoroborane (HBF_2_), we note that the B–H and B–F bonds lengths differ by just 0.001 Å from the CCSD(T)/aug-cc-pVTZ values, while a 0.1° difference is noted in the case of both the ∠HBF and ∠FBF values. A previous microwave spectroscopy study of HBF_2_ [38] provided B–F and B–H bond lengths of 1.311 ± 0.005 Å and 1.189 ± 0.010 Å, respectively. In the same study, the F–B–F bond angle was also reported as 118.3 ± 1°. Concerning the fluoroboryl radical (FHB•), the CCSD(T)/aVTZ level provides *r*(B–H) and *r*(B–F) distances that are 0.002 Å and 0.004 Å longer than those obtained at the B3LYP/A′VTZ level. We also note that, although not directly comparable with the *r_e_* values obtained by way of our calculations, a previous experimental study [39] reported the following geometric parameters for FHB•: *r*_0_(B–H) = 1.214(2) Å, *r*_0_(B–F) = 1.3034(5) Å, as well as an F–B–H angle of 120.7(1)°. In the case of boron trifluoride (BF_3_), the B–F bond length obtained at the B3LYP/A′VTZ level is only 0.001 Å longer than that obtained at the CCSD(T)/aug-cc-pVTZ level of theory. We note that the B3LYP/A′VTZ bond length (1.316 Å) differs by 0.009 Å from the experimentally reported value, *r_e_* = 1.3070(1) Å, which was obtained by infrared diode laser spectroscopy [40]. Finally, in the case of the difluoroboryl radical (F_2_B•), the B3LYP/A′VTZ level provides a B–F bond length that is 0.002 Å shorter than that obtained at the CCSD(T)/aug-cc-pVTZ level, while there is a 0.4° difference between these two methods with respect to ∠FBF.

### 2.2. Benchmark-Quality B–F BDEs via the W1w Thermochemical Protocol

Prior to presenting the B–F BDEs for the thirty molecules included in the BFBDE dataset, and subsequently discussing the effect that substituents play in governing the magnitude of these quantities, it is initially worthwhile commenting on the likely reliability of the W1w thermochemical protocol in the context of computing accurate B–F BDEs (i.e., with sub-kcal/mol accuracy) [41]. As the W1w thermochemical protocol: (i) makes use of the single-reference CCSD(T) method and (ii) does not include post-CCSD(T) corrections, we sought to elucidate whether such corrections are likely to result in significant additional contributions to the B–F BDEs.

To address the validity of the use of a single-reference method such as CCSD(T) for the calculation of the energies of the molecules of the species in this set, we have employed the *T*_1_ diagnostic proposed by Lee and Taylor [42], which has been shown to be a predictor of the quality of single-reference electron correlation methods. According to their findings, a *T*_1_ diagnostic for a given molecule of ≤0.02 indicates that a single-reference method should be sufficient for describing the electronic structure of the said molecule (i.e., such species are not likely to be subject to significant degrees of non-dynamical correlation). In this light, we note that the *T*_1_ diagnostics (calculated at the CCSD/A′VTZ level of theory) for nearly all the molecules considered in this study fall within this threshold of ≤0.02. The only exceptions to this were noted in the case of (HC_2_)HB• and (H_2_P)HB•, for which we computed *T*_1_ diagnostics of 0.03. 

We now turn our attention to considering the likely magnitude of any post-CCSD(T) contributions to the energies, which the W1w protocol does not consider (i.e., only going so far as the CCSD(T) level). To achieve this, we considered an energy-based diagnostic, namely the percentage of the atomization energy accounted for by parenthetically connected triple excitations, %TAE[(T)] [36,43]. This diagnostic has been shown to shed valuable insights concerning whether the inclusion of post-CCSD(T) corrections is necessary for computing reliable energies and was initially developed with regard to the computation of total atomization energies [44,45]. In particular, it has been demonstrated that for species with %TAE[(T)] ≤ 5%, post-CCSD(T) contributions are unlikely to exceed 2 kJ mol^−1^. The same diagnostic has also been employed previously for the purposes of validation of datasets of the BDEs for a range of other chemical bonds, including, for example, S–F [46], C–Cl [47], Al–H [48], and B–Cl bonds [49].

In the context of the molecules considered in the present study, we note that both the closed-shell parent precursor molecules, as well as the product radicals are all associated with %TAE[(T)] diagnostics that fall comfortably below the 5% threshold. In particular, (NC)_2_B• is associated with the largest %TAE[(T)] value of 3.8%. More broadly, we note that for approximately 92% of the species, the %TAE[(T)] diagnostics are ≤2.5%. Taking this finding into account, it would stand to reason that the inclusion of post-CCSD(T) contributions is unlikely to affect the B–F BDEs to a significant extent. Consequently, it is anticipated that the B–F BDEs reported in this study are expected to be within chemical accuracy (i.e., with deviations well below 4 kJ mol^−1^) from those that would be obtained at the full configuration interaction (FCI) infinite basis-set limit. 

It should be noted that for three boron fluorides (BF, BHF_2_, and BF_3_), high-level TAEs at the CCSDTQ5/CBS level of theory are available in the W4-17 database [41,44,45]. For these molecules, the post-CCSD(T) contributions amount to −0.25 (BF), −0.84 (BHF_2_), and −1.30 (BF_3_) kJ mol^−1^.

### 2.3. Effect of Substituents on the Strength of B–F Bonds toward Homolytic Dissociation

We now turn our attention to the set of 30 gas-phase homolytic B–F bond dissociation energies for a diverse array of fluoroborane-type molecules. This dataset is presented in Table 2, and we report BDEs at both 0 K (BDE_0_) and 298 K (BDE_298_), as well as non-relativistic bottom-of-the-well all-electron BDEs (BDE*_e_*), which will be used later in this article for the purposes of assessing a wide range of DFT functionals for their ability to compute B–F BDEs. We have also reported the equilibrium B–F bond lengths (*r*_B–F_) for each molecule. In addition, to facilitate an analysis of any stabilizing/destabilizing effects induced by the substituents in both the reactant closed-shell precursor and the product radicals, we have tabulated two other quantities. The so-called molecule stabilization enthalpy (MSE, Equation (2)) has been employed for considering the relative stabilizing/destabilizing effect of substituents in the closed-shell precursor molecules. In the context of the product radicals (R^1^R^2^B•), a similar approach has been taken, and thus we define the so-called radical stabilization enthalpy (RSE, Equation (3)).

R^1^R^2^B–F + H_2_B–H → R^1^R^2^B–H + H_2_B–F (2)

R^1^R^2^B–H + H_2_B• → R^1^R^2^B• + H_2_B–H (3)

Defined in this way, a positive MSE for a given system indicates that the substituents exert a relative stabilizing effect in R^1^R^2^B–F, while a negative value would indicate the existence of a relative destabilizing effect. In the context of the product radical, a positive RSE would indicate a relative destabilizing effect of the substituents in the substituted radical (R^1^R^2^B•), while a negative value would indicate a relative stabilizing effect. This type of approach has been employed previously for examining the effect of substituents in governing the strength of, for example, S–Cl [50], S–Br [51], C–Cl [52], and N–X (X = F and Cl) bonds [53]. 

Prior to considering the effect of substituents in governing the magnitude of the B–F BDEs, we begin by noting that the BDEs we have obtained for H_2_BF, HBF_2_, and BF_3_ (at 0 K) are in generally good agreement with those reported previously by Grant and Dixon [34]. In this regard, we note that our W1w values differ by 3.8 kJ mol^−1^ in the case of H_2_BF and 2.0 kJ mol^−1^ (in the case of both HBF_2_ and BF_3_). Furthermore, concerning the effect of substituents in governing the B–F bond distances, we note that these differ by as much as 0.049 Å. The shortest bond lengths were noted in the case of both (F_3_C)BHF and (NC)_2_BF (1.310 Å), while the longest B–F bond was observed in the case of (H_2_N)(H_2_B)BF (1.359 Å). 

Moving on to considering the broader effect of substituents in governing the B–F BDEs of the thirty molecules in the BFBDE dataset, we note that the BDEs (at 298 K) differ by up to 183.3 kJ mol^−1^ (Table 2). The results of our analysis clearly demonstrate that substitution with two –BH_2_ substituents (as in (H_2_B)_2_B–F) affords a molecule with the lowest BDE (545.9 kJ mol^−1^). This significant reduction appears to arise because of the combined effect of both a relative destabilizing effect in the parent fluoroborane derivative (MSE = −63.8 kJ mol^−1^) as well as a significant stabilizing effect in the product (H_2_B)_2_B• radical (RSE = −103.9 kJ mol^−1^). As noted previously in the context of B–Cl BDEs [49], an inspection of the spin densities (obtained at the B3LYP/A′VTZ level) of (H_2_B)_2_B• indicate a fair degree of delocalization of the unpaired electron onto the two substituent boron atoms (which are both associated with spin densities of 0.170). Such delocalization effects would contribute to this relatively high degree of stabilization of the product radical. In contrast, the highest B–F BDE is noted in the case of FHB–F (729.2 kJ mol^−1^), which is 15.7 kJ mol^−1^ higher in energy than that of the prototypical molecule H_2_B–F (BDE = 713.5 kJ mol^−1^). The fact that FHB–F has a higher BDE than H_2_B–F can be accounted for on the basis that the additional fluorine atom appears to induce a relative stabilizing effect in FHB–F (MSE = +17.1 kJ mol^−1^) that is of greater magnitude than that observed in the product radical (RSE = −1.4 kJ mol^−1^). Apart from FHB–F, we note that the second highest B–F BDE was noted in the case of BF_3_ (719.5 kJ mol^−1^). 

Of the monosubstituted species (i.e., R^1^HB–F), we note that the presence of a single formyl substituent appears to exert the most dramatic effect in terms of lowering the B–F BDE, even more so than substitution with a single –BH_2_ moiety. Thus, we find that the BDE of (HCO)HB–F (585.0 kJ mol^−1^) is 21.5 kJ mol^−1^ lower than that of (H_2_B)HB–F (606.5 kJ mol^−1^). Upon inspection of the MSEs and RSEs associated with these two species, it is evident that the lower BDE of (HCO)HB–F arises, for the most part, because the product radical is subject to a greater degree of stabilizing effects than observed in the case of (H_2_B)HB• (RSEs = −121.3 vs. −81.8 kJ mol^−1^, respectively). In fact, upon inspection of the geometry of the radical arising via dissociation of the B–F bond in (HCO)HB–F, we note that it adopts a three-membered ring structure (in which *r*(B–O) = 1.409 Å, *r*(C–O) = 1.437 Å, *r*(C = B) = 1.421 Å, and ∠COB = 59.9° at the B3LYP/A′VTZ level) which is subject to significant delocalization of the unpaired electron. Thus, for this radical, we have obtained spin densities (at the B3LYP/A′VTZ level of theory) of 0.307 on the O atom, 0.672 on the C atom, and only 0.037 on the B atom. For the remainder of the monosubstituted fluoroborane-type species, we note that for those substituents consisting of elements belonging to the second period, the BDEs (included in parentheses and expressed in kJ mol^−1^) increase in the order: HC(=O) (585.0) < BH_2_ (606.5) < HC≡C (688.0) < CN (696.7) < NH_2_ (706.0) < CH_3_ (715.8) < OH (717.0) < CF_3_ (718.7) < F (729.2).

Regarding those monosubstituted species containing third-period elements, we obtain the following trends in BDEs (included in parentheses and expressed in kJ mol^−1^): AlH_2_ (637.5) < PH_2_ (685.2) < SiH_3_ (686.6) < SH (687.3) < Cl (708.3). It is of interest to note that, apart from comparing BH_2_ and AlH_2_ (where substitution by –BH_2_ reduces the B–F BDE by 31.0 kJ mol^−1^ compared with the –AlH_2_ substituted molecule), it can be seen that for a given group (i.e., groups 14–17), substitution by elements belonging to the third period give rise to B–F BDEs that are smaller than those obtained in the case of substituents belonging to the second period. The following BDE reductions are noted in the case of each group: 29.2 (Group 14), 20.8 (Group 15), 29.7 (Group 16), and 20.9 kJ mol^−1^ (Group 17). In the case of comparing the effect of substitution by an -SiH_3_ vs. -CH_3_ substituent (i.e., where the atoms directly attached to the boron belong to Group 14), we note that the lower BDE of (H_3_Si)HB–F can be rationalized for on the basis that: (i) whereas (H_3_Si)HB–F is subject to a relative destabilizing effect (MSE = −9.4 kJ mol^−1^), the methyl-substituted species ((H_3_C)HB–F) is subject to a relative stabilizing effect (MSE = +6.4 kJ mol^−1^), and (ii) whereas both product radicals are subject to relative stabilizing effects (i.e., the RSE values are negative), the degree of relative stabilization in the case of (H_3_Si)HB• is larger (with the RSE of the silyl-substituted radical being 13.4 kJ mol^−1^ more exothermic than that of the methyl-substituted radical). In accounting for the fact that (H_2_P)HB–F has a lower BDE than that of (H_2_N)HB–F, we note that: (i) compared with (H_2_N)HB–F, the phosphine-substituted precursor has a more negative MSE (−18.1 vs. −11.3 kJ mol^−1^, respectively), and (ii) whereas there appears to be a relative destabilizing effect in (H_2_N)HB• (RSE = +3.7 kJ mol^−1^), the phosphine substituent in (H_2_P)HB• appears to induce a relative stabilizing effect (RSE = −10.2 kJ mol^−1^). Moving to a comparison of the Group 16 substituents, we note that the lower BDE of (HS)HB–F (687.3 kJ mol^−1^) vs. (OH)HB–F (717.0 kJ mol^−1^) evidently arises because of significant differences in the effect of substituents in the precursor fluorinated reactants, rather than any effects in the product radicals, given that both radicals are associated with RSE values that differ by just 1.0 kJ mol^−1^. In this regard, we note that whereas the MSE of (HO)HB–F is +8.1 kJ mol^−1^, the MSE of (HS)HB–F is −20.6 kJ mol^−1^. Similarly, we find that in comparing the Group 17 substituents, the larger BDE of FHB–F (729.2 kJ mol^−1^) compared with ClHB–F (708.3 kJ mol^−1^) also appears to arise because of a greater difference in the effect of substituents in the closed-shell precursor molecules (where the MSEs differ by 18.5 kJ mol^−1^), rather than differences in the product radicals (where the RSEs differ by just 2.4 kJ mol^−1^). 

### 2.4. Performance of DFT Methods for Computing B–F BDEs 

We now shift the focus of our attention to considering how a wide range of DFT methods performs in the context of their ability to provide accurate homolytic gas-phase B–F BDEs. To perform this assessment, we have performed the calculations in conjunction with the A′VQZ basis set (and using geometries obtained at the B3LYP/A′VTZ level), as this is expected to give results close to the basis set limit for each functional. It should be pointed out that in all cases, the B–F bond is homolytically cleaved to produce a fluorine atom and boron-centered radical in their doublet ground states. We also note that spin contamination does not represent a serious problem for the considered DFT methods, as indicated by <S^2^> ≈ 0.75 for the radicals at hand. Furthermore, as the reference set of BDEs, we have employed the full set of thirty non-relativistic bottom-of-the-well all-electron W1w BDEs as reference values (BDE*_e_*, Table 2). The results of this analysis are provided in Table 3. For each functional, we have reported the mean absolute deviations (MADs), mean deviations (MDs), largest deviations (LDs), and the number of outliers (NOs, which constitute the number of species with an absolute deviation from the W1w reference value larger than 10 kJ mol^−1^). 

**Figure 1:**
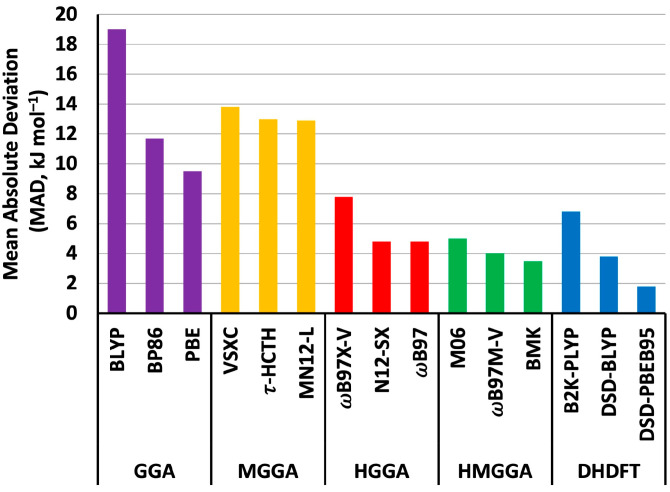
Mean absolute deviations (MADs) of the three best-performing functionals within each rung of Jacob′s Ladder (kJ mol^−1^).

We begin by offering a few general comments concerning the performance of the selected DFT methods for the computation of gas-phase homolytic B–F BDEs. First, the best-performing method appears to be DSD-PBEB95/A′VQZ, for which we compute an MAD of just 1.8 kJ mol^−1^ and an LD of 6.4 kJ mol^−1^. In contrast, the worst-performing method was shown to be the HGGA functional BH&HLYP, which afforded an unacceptably large MAD of 45.6 kJ mol^−1^ and an LD of 54.2 kJ mol^−1^. The poor performance of BH&HLYP in the computation of BDEs has been noted previously, for example, the computation of Al–H [48], B–Cl [49], and N–X (X = F, Cl, and Br) bonds [54,55]. Second, in all but four cases, it is evident that the functionals considered in this study tend to underestimate the BDEs (i.e., the MD values adopt negative values in most cases). Third, for the majority of functionals, the most problematic BDE to compute appears to be that of (NC)_2_B–F (molecule **10**), with approximately two-thirds of the functionals appearing to struggle with the computation of this bond dissociation energy. Prior to discussing the specific performance of the various functionals within each rung of Jacob’s Ladder (i.e., GGA, MGGA, HGGA, HMGGA, and DHDFT), Figure 1 provides a graphical representation of the MADs of the three best-performing functionals in each rung of Jacob′s Ladder.

Of the GGA functionals, we note that the MADs differ by up to 27.4 kJ mol^−1^, with revPBE offering the worst performance (MAD = 36.9 kJ mol^−1^ and LD = 54.6 kJ mol^−1^), while the best performing was PBE, with an MAD of 9.5 kJ mol^−1^, an LD of 26.4 kJ mol^−1^, and with 13 of the thirty BDEs having deviations from the W1w reference values ≥ 10 kJ mol^−1^. For all the GGA functionals, the most challenging molecule for which to calculate the BDE was (NC)_2_B–F (molecule **10**).

We now turn our attention to the performance of the MGGA functionals (for which we have considered the performance of seven such methods), in which the kinetic energy density is included. The best-performing MGGA functionals are MN12-L and *t*-HCTH, with MADs of 12.9 and 13.0 kJ mol^−1^, respectively. The worst performing MGGAs for the computation of B–F BDEs were shown to be *r*^2^SCAN and TPSS, with MADs of 25.4 and 28.7 kJ mol^−1^, respectively. Moving on to the hybrid GGA functionals (HGGA), we find that ωB97 and N12-SX both offer the lowest MADs (4.8 kJ mol^−1^ for both functionals), but we note that there is a significant difference in the magnitude of the LDs for these two functionals. In particular, whereas ωB97 has an LD of 15.4 kJ mol^−1^ (observed for the computation of the B–F BDE of molecule **3**, and which was the only BDE with a deviation ≥ 10 kJ mol^−1^**)**, the LD of N12-SX is observed in the case of molecule **10** and amounts to 20.7 kJ mol^−1^. The BH&HLYP functional, as mentioned previously, performed the worst of any of the functionals considered in this study, with an MAD of 45.6 kJ mol^−1^ and an LD of 54.2 kJ mol^−1^. The particularly poor performance of this HGGA functional sets it apart from the others in the HGGA family. In that regard, the next worst method was shown to be B3PW91 which attained an MAD of 27.5 kJ mol^−1^ (admittedly still offering unacceptable performance). The popular B3LYP functional attained an MAD of 22.2 kJ mol^−1^, which is better than its performance for the computation of gas-phase homolytic B–Cl BDEs, where it had attained an MAD of 30.1 kJ mol^−1^ in conjunction with the A′VQZ basis set [49]. Finally, we note that the ωB97-X functional offered an MAD that was 5.4 kJ mol^−1^ lower than that obtained with the related ωB97X-D functional.

Concerning the performance of the hybrid-meta-GGAs (HMGGAs), for which we have considered the performance of eleven such functionals, we note that these methods differ by up to 30.8 kJ mol^−1^ in terms of the MADs. In considering the worst performing of the HMGGAs, we note that TPSSh offered particularly poor performance (MAD = 34.3 kJ mol^−1^) given that the second worst performing method, namely PW6B95, attained an MAD of 12.7 kJ mol^−1^. The best performing HMGGA functional for the computation of B–F BDEs was BMK, and we note that the MAD of this functional (3.5 kJ mol^−1^) is similar to its MAD for the computation of B–Cl BDEs (4.0 kJ mol^−1^) [49]. The range-separated ωB97M-V functional performed only slightly worse, with an MAD of 4.0 kJ mol^−1^ and four BDEs that were ≥10 kJ mol^−1^ from the W1w reference values.

Of the ten double-hybrid DFT functionals that we have considered in this study, we note that two of these give MADs from the W1w reference values that are lower than 4 kJ mol^−1^. The DSD-PBEB95 functional, with an MAD of 1.8 kJ mol^−1^ and an LD of 6.4 kJ mol^−1^, attained the best overall performance of any of the functionals considered in this study. The next best performing DH functional was DSD-BLYP with an MAD of 3.8 kJ mol^−1^ and an LD of 10.0 kJ mol^−1^ (molecule **10**). Beyond these two better-performing DH functionals, the rest attained MADs between 6.8 kJ mol^−1^ (B2K-PLYP) and 25.4 kJ mol^−1^ (PBE0-DH).

As previous studies have shown that the inclusion of empirical dispersion corrections may improve the performance of the underlying DFT functionals for the computation of various BDEs [48,49,55], we have additionally surveyed the effect of the inclusion of the Becke–Johnson D3 dispersion correction for twelve functionals (Table 4). In addition to reporting the effect that inclusion of such corrections has on the MADs (∆MAD = MAD(DFT-D3) − MAD(DFT)), we have also looked at the effect on largest deviations (∆LD = LD(DFT-D3) − LD(DFT)). It follows that a negative ∆MAD or ∆LD value indicates that the inclusion of the D3BJ dispersion correction results in improved performance compared with that of the uncorrected functional.

The results of our analysis reveal that it is indeed the case that the inclusion of a Becke–Johnson D3 dispersion correction serves to improve the performance of all the functionals to which it has been appended for the computation of B–F BDEs. The largest performance improvement was noted in the case of revPBE, in which the MAD was reduced by 3.6 kJ mol^−1^ from that of the uncorrected functional (36.9 kJ mol^−1^), while there was a reduction in the LD by 3.8 kJ mol^−1^. The smallest performance enhancements in MAD were noted in the case of PW6B96 and BMK (∆MAD = −0.6 kJ mol^−1^), although we note that there was a larger decrease in the LD of BMK compared with that of PW6B95 (∆LD = −2.1 vs. −0.9 kJ mol^−1^, respectively). 

## 3. Computational Methods

In order to obtain equilibrium geometries for all of the molecules in this study, the B3LYP/A′VTZ level of theory (where A′V*n*Z denotes the use of cc-pV*n*Z basis sets for hydrogen, aug-cc-pV*n*Z for first-row elements, and aug-cc-pV(*n*+d)Z basis sets for second-row elements) has been utilized [56,57]. To confirm that the geometry of each molecule does indeed correspond to an equilibrium structure (i.e., a minimum) on the potential energy surface, we performed harmonic vibrational frequency calculations at the same level of theory, such as to ensure the absence of any imaginary frequencies. See Table S1 of the Supplementary Materials for additional details.

We then sought to perform calculations using the benchmark-quality W1w thermochemical protocol [35], which constitutes a layered extrapolation to the all-electron relativistic CCSD(T)/basis-set-limit, based on geometries obtained at the B3LYP/A′VTZ level of theory. We note that W1w theory has been found to consistently obtain BDEs and even total atomization energies (TAEs) with sub-chemical accuracy for systems that are not dominated by a strong multireference character [35,36,41,44,45,58]. With the intention of keeping this article self-contained, we will now briefly outline the protocol employed for obtaining the W1w energies. Initially, the underlying SCF/CBS energy was obtained using a two-point extrapolation of form E(*L*) = E_∞_ + A/*L*^5^ in conjunction with the A′VTZ and A′VQZ basis sets. Here, *L* is the highest angular momentum represented in the basis set, and E_∞_ and E(*L*) are the energies calculated with the finite basis set and at the infinite basis limit, respectively. Subsequently, we added the following corrections to the underlying SCF/CBS energy: (i) a correction for single and double excitations at the CCSD level (i.e., ∆CCSD), which is obtained using a two-point extrapolation of the form E(*L*) = E_∞_ + A/L^3.22^ in conjunction with the A′VTZ and A′VQZ basis sets), (ii) a correction for parenthetical triples excitations (i.e., ∆(T)), which is obtained using a two-point extrapolation of the form E(*L*) = E_∞_ + A/L^3.22^ in conjunction with the A′VDZ and A′VTZ basis sets), (iii) a core-valence correction (∆CV), which is computed as the difference between the all-electron CCSD(T)/MTsmall energies (with the exception of second-row elements, in which the 1*s* electrons are frozen) and the corresponding frozen core calculations, and finally (iv) a scalar relativistic correction (∆Rel.), which is obtained by way of Douglass–Kroll–Hess (DKH) calculations [59,60] and is computed as the difference in energy between a frozen-core DKH-CCSD(T)/MTsmall and frozen-core CCSD(T)/MTsmall calculation. The bottom-of-the-well non-relativistic all-electron W1w energy (i.e., W1w_AE,Rel._) is obtained as the sum of the SCF/CBS, ∆CCSD, ∆(T), ∆CV, and ∆Rel. components. In computing the W1w energy for fluorine atom, we have additionally included an atomic spin-orbit correction of 1.60 kJ mol^−1^ as taken from Ref. [35].

To obtain W1w energies at 0 K (i.e., W1w_0_) we added scaled ZPVE corrections, which were obtained at the B3LYP/A′VTZ level and scaled by 0.9884 (as recommended in the literature) [61] to the underlying W1w_AE,Rel._ energies. To obtain the enthalpies at 298 K (i.e., W1w_298_) we added H_vib_ corrections (obtained at the B3LYP/A′VTZ level of theory and scaled by 0.9987 as recommended in the literature) [61] to the W1w_0_ energies. 

We have also sought to identify more computationally efficient methods for the computation of homolytic gas-phase B–F BDEs, given that for larger molecules, the use of thermochemical protocols such as W1w may be computationally prohibitive. In this regard, we have assessed the performance of a plethora of DFT functionals for their ability to compute gas-phase homolytic B–F BDEs (in conjunction with the A′VQZ basis set and using geometries obtained at the B3LYP/A′VTZ level of theory). To perform this assessment, we have used, as reference values, the set of 30 W1w non-relativistic bottom-of-the-well BDEs (which do not include an atomic spin-orbit correction of 1.60 kJ mol^−1^ for fluorine atom). The DFT exchange-correlation functionals considered in this study, ordered by their rung on Jacob’s Ladder are the generalized gradient approximation (GGA) functionals: BLYP [62,63], B97-D [64], HCTH407 [65], PBE [66], revPBE [67], PB86 [63,68], and BPW91 [63,69], the meta-GGA functionals: TPSS [70], τ-HCTH [71], VSXC [72], MN12-L [73], MN15-L [74], *r*^2^SCAN [75], and B97M-V [76]; the hybrid-GGAs: BH&HLYP [77], B3LYP [62,78,79], B3PW91 [69,78], PBE0 [80], B97-1 [81], X3LYP [82], SOGGA11-X [83], APF [84], and the range-separated functionals ωB97 [85], ωB97X [85], N12-SX [86], CAM-B3LYP [87], ωB97X-V [88]; the hybrid-meta GGAs (HMGGAs): M06 [89], M06-2X [89], M08-HX [90], MN15 [74], BMK [91], TPSSh [92], τ-HCTHh [71], PW6B95 [93], and the range-separated functionals M11 [94], and ωB97M-V [95]; and the double hybrid (DH) functionals: B2-PLYP [96], B2K-PLYP [97], B2GP-PLYP [98], mPW2-PLYP [99], DSD-PBEP86 [100,101], DSD-BLYP [102], DSD-PBEB95 [100], PBE0-DH [103], PBEQI-DH [104] and PWPB95 [105]. For twelve functionals, for which Becke–Johnson D3 dispersion corrections are available, we have also assessed the performance of the inclusion of such corrections [106]. All DFT calculations for the open-shell species have been carried out using unrestricted formalism for the reference wave functions. All calculations have been performed using the Gaussian 16 program (Revision C.01) [107] and ORCA 5.0 programs [108,109]. 

## 4. Conclusions

In the present study, we have computed the gas-phase homolytic B–F BDEs of a set of thirty fluoroborane-type molecules at the CCSD(T)/CBS level of theory using the benchmark quality W1w thermochemical protocol. Using energy-based %TAE[(T)] diagnostics, we have validated the applicability of the W1w thermochemical protocol for the computation of gas-phase B–F BDEs. In this regard, we show that all the molecules considered in the study fall below the 5% threshold as suggested in the literature (the species in this set have %TAE[(T)] values that range from 0.2% in the case of H_2_B• to 3.8% in the case of (CN)_2_B•). The structural properties that affect the BDEs are examined in detail, and the homolytic BDEs are rationalized based on molecule stabilization enthalpies (MSEs) and radical stabilization enthalpies (RSEs). The B–F BDEs of the molecules in this set differ by up to as much as 183.3 kJ mol^−1^, with (H_2_B)_2_BF having the lowest BDE (545.9 kJ mol^−1^) and HBF_2_ having the largest (729.2 kJ mol^−1^). Apart from substituents belonging to Group 13 (i.e., BH_2_ and AlH_2_), we note that for the rest (Groups 14–17), substitution with third-period elements give rise to lower B–F BDEs than those molecules containing substituents belonging to the second period. We have additionally assessed the performance of a wide range of DFT methods, in conjunction with the A′VQZ basis set, against the complete set of thirty gas-phase B–F BDEs obtained at the W1w level of theory. We find that, of all the methods examined, the double-hybrid functional DSD-PBEB95 offers the best performance, with a mean absolute deviation (MAD) of just 1.8 kJ mol^−1^ and the largest deviation (LD) of just 6.4 kJ mol^−1^. We have also shown that the inclusion of a Becke–Johnson D3 dispersion correction is advantageous, resulting in generally modest improvements in performance relative to that obtained with the uncorrected functionals. In this light, we observe improvements in MADs by amounts ranging from 0.6 kJ mol^−1^ (in the case of both PW6B95 and BMK) to 3.6 kJ mol^−1^ in the case of revPBE.

## Figures and Tables

**Table 1:** Comparison of Various Geometric Parameters Obtained at the B3LYP/A′VTZ Level versus those Reported Previously at the CCSD(T)/aug-cc-pVTZ Level of Theory (denoted as CCSD(T)/aVTZ) for a Set of Fluoroborane-Type Molecules and Radicals (Bond Lengths Expressed in Angstrom (Å) Units and Bond Angles in Degrees).

Molecule	Level	*r*(B–H)	*r*(B–F)	∠HBF	∠FBF
H_2_BF	B3LYP/A′VTZ	1.191	1.322	117.8	–
	CCSD(T)/aVTZ *^a^*	1.193	1.325	117.8	–
HBF_2_	B3LYP/A′VTZ	1.185	1.318	120.9	118.1
	CCSD(T)/aVTZ *^a^*	1.186	1.319	121.0	118.0
FHB•	B3LYP/A′VTZ	1.201	1.305	121.1	–
	CCSD(T)/aVTZ *^a^*	1.203	1.309	121.0	–
BF_3_	B3LYP/A′VTZ	–	1.316	–	120.0
	CCSD(T)/aVTZ *^a^*	–	1.315	–	120.0
F_2_B•	B3LYP/A′VTZ	–	1.310	–	121.4
	CCSD(T)/aVTZ *^a^*	–	1.312	–	121.0

*^a^* Values taken from Reference [34].

**Table 2:** Gas-Phase Homolytic B–F Bond Dissociation Enthalpies (BDEs) for Boron Fluorides (R^1^R^2^B–F), Molecule Stabilization Enthalpies (MSEs), Radical Stabilization Enthalpies (RSEs) and Equilibrium B–F Bond Lengths for All Molecules in the BFBDE Dataset (All Energies Expressed in kJ mol^−1^).

Molecule	R^1^	R^2^	BDE_0_	BDE_298_	BDE*_e_*	MSE	RSE	*r*_B–F_ (Å)
**1**	BH_2_	BH_2_	542.4	545.9	554.6	−63.8	−103.9	1.352
**2**	AlH_2_	AlH_2_	576.2	578.1	585.2	−56.0	−79.5	1.347
**3**	HC(=O)	H	581.5	585.0	597.2	−7.3	−121.3	1.321
**4**	NH_2_	BH_2_	594.6	598.5	609.1	−19.5	−95.6	1.359
**5**	BH_2_	H	601.9	606.5	618.3	−25.2	−81.8	1.335
**6**	AlH_2_	H	634.0	637.5	648.4	−17.5	−58.5	1.336
**7**	SiH_3_	SiH_3_	652.0	655.7	665.3	−23.4	−34.4	1.333
**8**	PH_2_	PH_2_	657.5	661.5	671.0	−29.9	−22.2	1.335
**9**	SH	SH	668.0	672.3	682.2	−32.8	−8.4	1.332
**10**	CN	CN	680.3	684.7	696.9	−20.6	−8.2	1.310
**11**	PH_2_	H	680.7	685.2	697.6	−18.1	−10.2	1.328
**12**	SiH_3_	H	682.1	686.6	700.1	−9.4	−17.5	1.327
**13**	SH	H	682.6	687.3	699.7	−20.6	−5.6	1.328
**14**	HC≡C	H	682.4	688.0	704.2	−8.6	−16.9	1.327
**15**	Cl	Cl	691.2	695.7	706.0	−16.4	−1.4	1.315
**16**	CN	H	691.6	696.7	712.0	−11.3	−5.5	1.314
**17**	NH_2_	H	700.9	706.0	719.1	−11.3	3.7	1.343
**18**	NH_2_	NH_2_	703.2	708.0	720.0	−14.4	8.9	1.356
**19**	Cl	H	703.1	708.3	722.0	−1.4	−3.8	1.315
**20**	Cl	F	705.3	710.1	721.1	−9.2	5.7	1.315
**21**	SiH_3_	F	706.1	710.3	721.4	19.1	−22.4	1.325
**22**	OH	OH	705.8	711.0	722.3	−7.7	5.1	1.338
**23**	H	H	707.5	713.5	731.0	0.0	0.0	1.322
**24**	CH_3_	H	710.9	715.8	731.3	6.4	−4.1	1.332
**25**	CH_3_	CH_3_	711.8	716.1	729.3	8.7	−6.1	1.344
**26**	OH	H	711.7	717.0	730.6	8.1	−4.6	1.337
**27**	NH_2_	F	712.7	717.6	729.1	−7.8	11.9	1.335
**28**	CF_3_	H	714.2	718.7	733.3	−3.4	8.5	1.310
**29**	F	F	714.5	719.5	730.9	−9.6	15.6	1.316
**30**	F	H	723.7	729.2	743.8	17.1	−1.4	1.318

**Table 3:** Performance of a Diverse Array of DFT Methods for the Computation of Gas-Phase Homolytic B–F Bond Dissociation Energies in Conjunction with the A′VQZ Basis Set (All Energies Are Expressed in kJ mol^−1^).

Class *^a^*	Method	MAD	MD	LD *^c^*	NO
GGA	revPBE	36.9	−36.9	54.6 (**10**)	30
	BPW91	22.5	−22.5	40.8 (**10**)	29
	HCTH407	20.4	−20.4	40.9 (**10**)	27
	B97-D	19.1	−19.1	41.5 (**10**)	27
	BLYP	19.0	−19.0	40.6 (**10**)	25
	BP86	11.7	−11.3	30.3 (**10**)	19
	PBE	9.5	−7.9	26.4 (**10**)	13
MGGA	TPSS	28.7	−28.7	43.4 (**10**)	30
	*r*^2^SCAN	25.4	−25.4	42.4 (**10**)	30
	MN15-L	20.2	−20.2	39.2 (**4**)	30
	B97M-V	16.0	−16.0	39.0 (**10**)	23
	VSXC	13.8	−13.8	37.1 (**10**)	17
	τ-HCTH	13.0	−12.8	35.1 (**10**)	19
	MN12-L	12.9	−12.9	37.0 (**10**)	15
HGGA	BH&HLYP	45.6	−45.6	54.2 (**10**)	30
	B3PW91	27.5	−27.5	40.5 (**10**)	30
	APF	26.8	−26.8	38.8 (**10**)	30
	PBE0	26.3	−26.3	37.6 (**10**)	30
	APF-D	23.6	−23.6	35.5 (**10**)	30
	B3LYP	22.2	−22.2	37.7 (**10**)	30
	X3LYP	20.4	−20.4	35.5 (**10**)	30
	SOGGA11-X	19.0	−19.0	27.6 (**10**)	30
	ωB97X-D *^b^*	16.7	−16.7	23.6 (**10**)	29
	B97-1	12.7	−12.7	27.2 (**10**)	20
	ωB97-X *^b^*	11.3	−11.3	16.2 (**10**)	20
	CAM-B3LYP *^b^*	8.5	−8.5	16.7 (**10**)	10
	ωB97X-V *^b^*	7.8	−7.8	15.5 (**3**)	7
	N12-SX *^b^*	4.8	−4.2	20.7 (**10**)	3
	ωB97 *^b^*	4.8	−4.8	15.4 (**3**)	1
HMGGA	TPSSh	34.3	−34.3	46.3 (**10**)	30
	PW6B95	12.7	−12.7	24.4 (**3**)	19
	τ-HCTHh	9.1	−9.0	26.4 (**10**)	13
	M05-2X	9.0	+8.4	14.2 (**29**)	11
	M06-2X	7.3	−7.1	15.3 (**3**)	7
	M08-HX	7.3	−7.3	12.1 (**23**)	8
	MN15	6.2	+2.3	16.4 (**3**)	5
	M11 *^b^*	5.8	+0.4	18.9 (**1**)	5
	M06	5.0	−4.5	18.5 (**10**)	4
	ωB97M-V *^b^*	4.0	−3.8	14.1 (**4**)	4
	BMK	3.5	−2.6	15.7 (**3**)	2
DHDFT	PBE0-DH	25.4	−25.4	32.4 (**3**)	30
	PBE-QIDH	13.8	−13.8	20.3 (**10**)	30
	mPW2-PLYP	11.0	−11.0	16.6 (**10**)	21
	B2-PLYP	10.6	−10.6	16.5 (**10**)	17
	DSD-PBEP86	9.3	−9.3	13.2 (**29**)	14
	PWPB95	8.6	−8.6	14.5 (**3**)	8
	B2GP-PLYP	8.4	−8.4	11.8 (**29**)	5
	B2K-PLYP	6.8	−6.8	9.9 (**29**)	0
	DSD-BLYP	3.8	+3.8	10.0 (**10**)	0
	DSD-PBEB95	1.8	−1.1	6.4 (**3**)	0

*^a^* GGA, generalized gradient approximation; MGGA, meta-GGA; HGGA, hybrid-GGA; HMGGA, hybrid-meta-GGA; DH, double hybrid). *^b^* Range separated XC functional. *^c^* The molecule giving rise to the largest deviation is included in parentheses.

**Table 4:** Effect of the Inclusion of the Becke–Johnson D3 Dispersion Correction on the MADs and LDs of Selected Functionals (All Energies Are Expressed in kJ mol^−1^).

Class	Functional	∆MAD *^a^*	∆LD *^b^*
GGA	revPBE	−3.6	−3.8
	BLYP	−2.7	−3.1
	BP86	−1.6	−2.4
	PBE	−1.0	−1.6
MGGA	TPSS	−1.8	−2.0
HGGA	B3PW91	−2.3	−2.6
	PBE0	−1.1	−1.4
	B3LYP	−2.3	−2.5
	CAM-B3LYP	−1.0	−1.3
HMGGA	PW6B95	−0.6	−0.9
	BMK	−0.6	−2.1
DH	B2-PLYP	−1.0	−1.1

*^a^* ∆MAD = MAD (DFT-D3) − MAD (DFT). *^b^* ∆LD = LD (DFT-D3) − LD (DFT).

## Data Availability

Not applicable.

## Supplementary Materials

The following supporting information can be downloaded at: https://www.mdpi.com/article/10.3390/molecules28155707/s1. Optimized geometries (in Cartesian coordinates) of all molecules investigated in this study (obtained at the B3LYP/A′VTZ level of theory) are provided in Table S1. In addition, the raw W1w energies (including the various individual energies that led to the computation of these quantities for each molecule), as well as the raw DFT energies used in assessing the performance of such methods for the computation of gas-phase B–F BDEs have been included as an Excel spreadsheet.
