# Supplementary material for: A Systematic Exploration of B–F Bond Dissociation Enthalpies of Fluoroborane-Type Molecules at the CCSD(T)/CBS Level
Source: Molecules. 2023 Jul 28;28(15):5707. doi: 10.3390/molecules28155707 (PMC10420309; doi:10.3390/molecules28155707)
Supplement: Supplementary file 1 [file molecules-28-05707-s001.zip › BF_Molecules_SI.pdf]

Supporting Information for

A Systematic Exploration of B–F Bond Dissociation Enthalpies of Fluoroborane-Type Molecules at the CCSD(T)/CBS Level

Robert J. O'Reilly* and Amir Karton*

School of Science and Technology, University of New England, Armidale, NSW 2351, Australia.

*E-Mail Addresses: roreill6@une.edu.au (RJO); amir.karton@une.edu.au (AK).

Table S1. Equilibrium geometries of all molecules obtained at the B3LYP/A'VTZ level of theory (in Cartesian Coordinates)

$(H_2B)_2B$–F (Molecule **1**)

B	0.000000	0.000000	0.019642
B	-0.000000	1.473829	-0.794560
F	0.000000	0.000000	1.371404
B	-0.000000	-1.473829	-0.794560
H	1.013976	2.009628	-1.123812
H	-1.013976	2.009628	-1.123812
H	-1.013976	-2.009628	-1.123812
H	1.013976	-2.009628	-1.123812

$(H_2B)_2B$• (Molecule **1** – Radical)

B	0.000000	0.000000	0.000000
B	0.000000	-0.000000	1.601495
B	0.000000	-0.000000	-1.601495
H	0.000000	1.020201	2.218983
H	-0.000000	-1.020201	2.218983
H	-0.000000	-1.020201	-2.218983
H	0.000000	1.020201	-2.218983

$(H_2Al)_2B$–F (Molecule **2**)

B	-0.000000	0.000000	0.444361
Al	-0.000000	1.936448	-0.552182
F	-0.000000	0.000000	1.791674
Al	-0.000000	-1.936448	-0.552182
H	1.357318	2.630013	-0.997537
H	-1.357318	2.630013	-0.997537
H	-1.357318	-2.630013	-0.997537
H	1.357318	-2.630013	-0.997537

$(H_2Al)_2B$• (Molecule **2** – Radical)

B	0.000000	0.000000	0.000000
Al	-0.000000	0.000000	2.080003
Al	0.000000	0.000000	-2.080003
H	-0.000000	1.375859	2.862801
H	-0.000000	-1.375859	2.862801
H	-0.000000	-1.375859	-2.862801
H	0.000000	1.375859	-2.862801

(HCO)HB–F (Molecule **3**)

B	0.622672	0.481379	0.000008
F	1.765726	-0.179962	-0.000007
C	-0.733186	-0.376935	0.000009
H	0.631711	1.669207	0.000020
O	-1.820448	0.161656	-0.000009
H	-0.673898	-1.488090	0.000023

(HCO)HB• (Molecule **3** – Radical)

B	0.742598	-0.431245	-0.000002
C	-0.677752	-0.467361	0.000090
H	1.867304	-0.766038	-0.000038
O	0.029053	0.783733	-0.000082
H	-1.746204	-0.543434	0.000165

$(NH_2)(BH_2)B$–F (Molecule **4**)

B	0.000000	0.019792	0.000000
F	1.229325	-0.559492	0.000000
B	-0.046070	1.712764	0.000000
N	-1.099217	-0.846899	0.000000
H	-0.046746	2.345612	1.011387
H	-0.046746	2.345612	-1.011387
H	-0.989797	-1.849025	0.000000
H	-2.055768	-0.541258	0.000000

$(NH_2)(BH_2)B$• (Molecule **4** – Radical)

B	0.000000	0.000000	-0.055030
B	0.000000	0.000000	-1.629416
N	-0.000000	-0.000000	1.309120
H	-0.000000	-1.027332	-2.235699
H	0.000000	1.027332	-2.235699
H	-0.842448	0.000000	1.864890
H	0.842448	-0.000000	1.864890

$(H_2B)HB$–F (Molecule **5**)

B	0.000000	0.488245	0.000000
B	-1.403474	-0.422122	0.000000
F	1.216209	-0.061396	-0.000000
H	-0.026986	1.690799	0.000000
H	-1.950763	-0.734426	1.013059
H	-1.950763	-0.734426	-1.013059

$(H_2B)HB$• (Molecule **5** – Radical)

B	-0.000000	0.000000	0.843892
B	0.000000	-0.000000	-0.716177
H	-0.000000	0.000000	2.019316
H	0.000000	1.024832	-1.328944
H	-0.000000	-1.024832	-1.328944

$(H_2Al)HB$–F (Molecule **6**)

B	0.000000	0.958644	0.000000
F	-1.307989	1.228430	0.000000
Al	0.699443	-1.083858	0.000000
H	0.701509	1.936404	0.000000
H	0.988818	-1.847672	1.358466
H	0.988818	-1.847672	-1.358466

$(H_2Al)HB$• (Molecule **6** – Radical)

B	-0.000000	-0.000000	-1.470164
Al	0.000000	0.000000	0.566029
H	-0.000000	-0.000000	-2.652103
H	0.000000	1.386819	1.322271
H	-0.000000	-1.386819	1.322271

$(SiH_3)_2B–F$ (Molecule **7**)

Si	0.000000	1.810370	-0.496525
B	0.000000	0.000000	0.461939
H	1.397882	2.111256	-0.903252
H	-0.495107	2.919523	0.354055
H	-0.819762	1.741144	-1.731200
F	0.000000	0.000000	1.794865
Si	-0.000000	-1.810370	-0.496525
H	0.819762	-1.741144	-1.731200
H	-1.397882	-2.111256	-0.903252
H	0.495107	-2.919523	0.354055

$(SiH_3)_2B$• (Molecule **7** – Radical)

Si	0.000000	1.963146	0.000000
B	0.000000	0.000000	0.000000
H	1.447988	2.309125	0.000000
H	-0.629162	2.574106	1.197905
H	-0.629162	2.574106	-1.197905
Si	-0.000000	-1.963146	0.000000
H	0.629162	-2.574106	-1.197905
H	-1.447988	-2.309125	0.000000
H	0.629162	-2.574106	1.197905

$(PH_2)_2B–F$ (Molecule **8**)

B	0.017624	0.390797	-0.000000
P	-0.086553	-0.504777	1.706398
P	-0.086553	-0.504777	-1.706398
F	-0.086553	1.721557	-0.000000
H	0.776705	0.364504	2.412543
H	0.867021	-1.516857	1.458846
H	0.776705	0.364504	-2.412543
H	0.867021	-1.516857	-1.458846

$(PH_2)_2B$• (Molecule **8** – Radical)

B	-0.079040	0.431087	0.000000
P	-0.079040	-0.097731	1.760635
P	-0.079040	-0.097731	-1.760635
H	0.201371	1.106690	2.441882
H	1.181830	-0.718450	1.947211
H	0.201371	1.106690	-2.441882
H	1.181830	-0.718450	-1.947211

$(SH)_2B–F$ (Molecule **9**)

B	0.000000	0.366587	0.000000
F	-0.017069	1.698536	0.000000
S	1.632210	-0.422789	-0.000000
S	-1.549922	-0.572850	0.000000
H	1.193981	-1.691042	-0.000000
H	-2.356964	0.501499	0.000000

$(SH)_2B$• (Molecule **9** – Radical)

B	0.000000	-0.000000	0.623661
S	-0.000000	1.588943	-0.157347
S	-0.000000	-1.588943	-0.157347
H	0.000000	2.339732	0.958391
H	-0.000000	-2.339732	0.958391

$(NC)_2B–F$ (Molecule **10**)

B	0.000000	0.000000	0.404768
F	0.000000	0.000000	1.715173
C	0.000000	1.333666	-0.355897
C	-0.000000	-1.333666	-0.355897
N	0.000000	-2.325031	-0.942117
N	0.000000	2.325031	-0.942117

$(NC)_2B$• (Molecule **10** – Radical)

B	0.000000	0.000000	0.436087
C	-0.000000	1.403745	0.040098
C	-0.000000	-1.403745	0.040098
N	0.000000	-2.543556	-0.190115
N	0.000000	2.543556	-0.190115

$(PH_2)HB–F$ (Molecule **11**)

B	0.679183	0.547510	0.025415
P	-1.121984	-0.083463	-0.116181
F	1.691565	-0.311561	0.005413
H	0.941800	1.711236	0.017677
H	-1.697349	0.813087	0.811362
H	-1.034702	-1.205884	0.737887

$(PH_2)HB$• (Molecule **11** – Radical)

B	1.334572	-0.174983	0.070710
P	-0.468731	0.016370	-0.110564
H	2.279327	0.529912	0.000791
H	-0.881514	1.126963	0.663087
H	-1.039711	-1.027519	0.641038

$(SiH_3)HB–F$ (Molecule **12**)

B	0.000000	0.962567	0.000000
Si	0.604844	-0.983429	0.000000
H	0.761556	1.886497	0.000000
F	-1.289811	1.274179	0.000000
H	1.448913	-1.224977	1.197777
H	1.448913	-1.224977	-1.197777
H	-0.518909	-1.948976	0.000000

(SiH$_3$)HB• (Molecule **12** – Radical)

B	-1.480374	0.139641	-0.015591
Si	0.489634	-0.013087	-0.003281
H	-2.491282	-0.474498	-0.020139
H	1.067029	-1.320889	-0.413903
H	0.874575	0.241154	1.410631
H	1.096679	1.039250	-0.852702

(HS)HB–F (Molecule **13**)

B	0.000000	0.814677	0.000000
F	1.315098	0.998009	0.000000
S	-0.721714	-0.830891	0.000000
H	-0.722900	1.756884	0.000000
H	0.434441	-1.518087	0.000000

(HS)HB• (Molecule **13** – Radical)

B	-0.015780	-1.270891	-0.000000
S	-0.015780	0.475030	0.000000
H	-0.980597	-1.956027	-0.000000
H	1.311968	0.710002	0.000000

(HC$_2$)HB–F (Molecule **14**)

B	0.623397	-0.608253	0.000000
C	-0.000000	0.776798	0.000000
F	-0.158784	-1.679870	0.000000
H	1.800456	-0.776745	0.000000
C	-0.441409	1.898630	0.000000
H	-0.839935	2.884266	0.000000

(HC$_2$)HB• (Molecule **14** – Radical)

B	-1.441567	-0.023639	0.000000
C	0.000000	0.008173	0.000000
H	-2.386606	0.674154	0.000000
C	1.219134	-0.076557	0.000000
H	2.279640	-0.145654	0.000000

Cl$_2$B–F (Molecule **15**)

B	-0.000000	-0.000000	0.356095
Cl	0.000000	-1.526298	-0.494586
Cl	0.000000	1.526298	-0.494586
F	-0.000000	-0.000000	1.670607

Cl$_2$B• (Molecule **15** – Radical)

B	-0.000000	0.000000	0.683183
Cl	0.000000	1.538675	-0.100468
Cl	-0.000000	-1.538675	-0.100468

(NC)HB–F (Molecule **16**)

B	0.598890	-0.595542	-0.000000
H	1.770159	-0.771591	-0.000000
C	0.000000	0.827613	0.000000
F	-0.223255	-1.620971	0.000000
N	-0.393616	1.910338	0.000000

(NC)HB• (Molecule **16** – Radical)

B	0.047597	-1.387754	-0.000000
H	-0.716262	-2.279154	-0.000000
C	0.000000	0.083517	0.000000
N	0.068326	1.245260	0.000000

(NH$_2$)HB–F (Molecule **17**)

B	0.000000	0.495631	0.000000
N	1.117693	-0.335944	0.000000
F	-1.225735	-0.054188	0.000000
H	0.097591	1.681719	0.000000
H	2.056685	0.020252	0.000000
H	1.053486	-1.340830	0.000000

(NH$_2$)HB• (Molecule **17** – Radical)

B	0.064548	0.854536	0.000000
N	0.064548	-0.522381	-0.000000
H	-0.904207	1.545247	0.000000
H	-0.776351	-1.085439	-0.000000
H	0.905984	-1.075821	-0.000000

(NH$_2$)$_2$B–F (Molecule **18**)

B	0.000000	0.000000	0.016417
N	-0.000000	1.252823	-0.637442
N	-0.000000	-1.252823	-0.637442
F	0.000000	0.000000	1.371977
H	-0.000000	2.112823	-0.119547
H	-0.000000	1.371790	-1.633296
H	-0.000000	-2.112823	-0.119547
H	-0.000000	-1.371790	-1.633296

(NH$_2$)$_2$B• (Molecule **18** – Radical)

B	0.000000	0.000000	0.522727
N	-0.000000	1.258572	-0.092066
N	-0.000000	-1.258572	-0.092066
H	-0.000000	2.114733	0.430750
H	-0.000000	1.401608	-1.093107
H	-0.000000	-2.114733	0.430750
H	-0.000000	-1.401608	-1.093107

ClHB–F (Molecule **19**)

B	0.000000	0.787776	0.000000
H	-0.722007	1.725645	0.000000
Cl	-0.648339	-0.838268	0.000000
F	1.304862	0.954004	0.000000

ClHB• (Molecule **19** – Radical)

B	0.042898	1.239614	0.000000

H	-0.943750	1.900668	0.000000
Cl	0.042898	-0.476396	0.000000

ClFB–F (Molecule **20**)

B	0.000000	0.000000	-0.442568
F	0.000000	-1.133067	-1.109661
F	0.000000	1.133067	-1.109661
Cl	-0.000000	-0.000000	1.305102

ClFB• (Molecule **20** – Radical)

B	0.000000	0.815034	0.000000
F	1.283568	1.043438	0.000000
Cl	-0.679536	-0.792124	0.000000

(SiH_3)FB–F (Molecule **21**)

B	-0.587849	-0.000002	-0.008824
F	-1.288826	1.124186	0.000778
F	-1.288845	-1.124178	0.000778
Si	1.449239	-0.000005	-0.001841
H	1.968730	1.210455	-0.675728
H	1.968744	-1.210671	-0.675345
H	1.911463	0.000226	1.406966

(SiH_3)FB• (Molecule **21** – Radical)

B	0.000000	-0.968049	-0.000000
F	1.209241	-1.457228	-0.000000
Si	-0.603605	0.975047	0.000000
H	0.464694	2.009370	0.000000
H	-1.448699	1.147630	1.204223
H	-1.448699	1.147630	-1.204223

$(OH)_2$B–F (Molecule **22**)

B	-0.016896	0.013596	0.000000
F	-1.208613	-0.595118	0.000000
O	0.000000	1.369685	-0.000000
O	1.135373	-0.717510	-0.000000
H	0.887430	1.738540	-0.000000
H	0.991576	-1.667853	0.000000

$(HO)_2$B• (Molecule **22** – Radical)

B	-0.000000	0.000000	0.475005
O	-0.000000	1.174077	-0.195073
O	-0.000000	-1.174077	-0.195073
H	0.000000	1.947343	0.373072
H	-0.000000	-1.947343	0.373072

H_2B–F (Molecule **23**)

B	0.000000	0.000000	-0.674175
H	0.000000	1.054009	-1.229789
H	-0.000000	-1.054009	-1.229789
F	-0.000000	-0.000000	0.647828

H_2B• (Molecule **23** – Radical)

B	0.000000	-0.000000	0.144082
H	0.000000	-1.072896	-0.360204
H	0.000000	1.072896	-0.360204

(CH_3)HB–F (Molecule **24**)

B	0.000000	0.520886	0.000000
C	1.147833	-0.529047	0.000000
F	-1.273676	0.130449	0.000000
H	0.183647	1.702305	0.000000
H	0.802734	-1.562696	0.000000
H	1.794855	-0.371899	0.869235
H	1.794855	-0.371899	-0.869235

(CH_3)HB• (Molecule **24** – Radical)

B	-0.931407	-0.212332	-0.013935
C	0.588686	0.020263	-0.009905
H	-1.804625	0.596997	0.007028
H	1.205108	-0.852678	-0.223804
H	0.834944	0.350209	1.011582
H	0.889491	0.845550	-0.665701

$(CH_3)_2$B–F (Molecule **25**)

C	0.000000	1.391295	-0.657572
B	0.000000	0.000000	0.061191
H	1.041728	1.675286	-0.852387
H	-0.434371	2.184052	-0.047529
H	-0.494810	1.368093	-1.629484
F	0.000000	0.000000	1.404856
C	-0.000000	-1.391295	-0.657572
H	0.494810	-1.368093	-1.629484
H	-1.041728	-1.675286	-0.852387
H	0.434371	-2.184052	-0.047529

$(CH_3)_2$B• (Molecule **25** – Radical)

C	-0.000000	1.401504	-0.097076
B	0.000000	0.000000	0.557630
H	1.058696	1.676112	-0.213156
H	-0.452829	2.189519	0.504723
H	-0.437665	1.418697	-1.103186
C	-0.000000	-1.401504	-0.097076
H	0.437665	-1.418697	-1.103186
H	-1.058696	-1.676112	-0.213156
H	0.452829	-2.189519	0.504723

(OH)HB–F (Molecule **26**)

B	-0.000000	0.491508	0.000000
F	-1.159247	-0.175072	0.000000
O	1.170739	-0.177737	0.000000

H	-0.008353	1.677353	0.000000
H	1.075663	-1.137347	0.000000

(HO)HB• (Molecule **26** – Radical)

B	0.010332	-0.788454	0.000000
O	0.010332	0.548228	0.000000
H	-1.013386	-1.406133	0.000000
H	0.879066	0.962577	0.000000

(NH_2)FB–F (Molecule **27**)

B	-0.000000	-0.000000	0.037017
F	0.000000	1.141378	0.728917
F	-0.000000	-1.141378	0.728917
N	0.000000	0.000000	-1.358494
H	0.000000	0.846794	-1.898065
H	0.000000	-0.846794	-1.898065

(NH_2)FB• (Molecule **27** – Radical)

B	0.534341	-0.016646	0.000000
F	0.000000	-1.232736	-0.000000
N	-0.229749	1.141119	0.000000
H	0.177633	2.058651	0.000000
H	-1.241095	1.131369	0.000000

(CF_3)HB–F (Molecule **28**)

B	-0.548671	-1.148583	0.000000
F	0.361528	-2.090283	0.000000
H	-1.702700	-1.419996	0.000000
C	-0.063763	0.391077	0.000000
F	1.272330	0.556237	0.000000
F	-0.548671	1.034604	1.091866
F	-0.548671	1.034604	-1.091866

(CF_3)HB• (Molecule **28** – Radical)

B	-1.366663	0.062121	-0.969555
H	-2.452919	-0.211658	-0.594072
C	-0.045453	-0.001342	-0.086714
F	1.112614	0.147908	-0.760620
F	0.057016	-1.167595	0.599311
F	-0.107524	1.009588	0.823769

F_2B–F (Molecule **29**)

B	0.000000	0.000000	0.000000
F	0.000000	1.315759	-0.000000
F	1.139481	-0.657880	-0.000000
F	-1.139481	-0.657880	0.000000

F_2B• (Molecule **29** – Radical)

B	0.000000	-0.000000	0.501784
F	0.000000	-1.142524	-0.139384
F	0.000000	1.142524	-0.139384

FHB–F (Molecule **30**)

B	0.000000	-0.000000	0.458986
F	0.000000	-1.130339	-0.218838
F	0.000000	1.130339	-0.218838
H	0.000000	-0.000000	1.644149

FHB• (Molecule **30** – Radical)

B	0.068568	0.741459	0.000000
F	0.068568	-0.563197	0.000000
H	-0.959951	1.361476	0.000000
